# The mediating role of resilience and interaction anxiousness in the effects of physical activity on mobile phone addiction among Chinese college students

**DOI:** 10.3389/fpsyt.2024.1378438

**Published:** 2024-04-24

**Authors:** Jinlong Wu, Wen Xiao, Bowen Liu, Jingxuan Yu, Kangyong Zheng, Qiuqiong Shi, Zhanbing Ren

**Affiliations:** ^1^ College of Physical Education, Southwest University, Chongqing, China; ^2^ College of Physical Education, Shenzhen University, Shenzhen, China; ^3^ Department of Rehabilitation Sciences, The Hong Kong Polytechnic University, Hong Kong, Hong Kong SAR, China; ^4^ Laboratory for Artificial Intelligence in Design, Hong Kong, Hong Kong SAR, China

**Keywords:** physical activity, resilience, interaction anxiousness, mobile phone addiction, college students

## Abstract

**Background:**

Recent studies have shown that regular physical activity (PA) can positively influence mobile phone addiction (MPA) behaviors in college students. However, it remains unknown whether this effect is mediated by other factors. Evidence suggests that resilience and interaction anxiousness may be candidate mediators that partly explain the positive effect of PA on MPA. This study aims to explore the impact of PA on MPA through a mediation model, and the role of resilience and interaction anxiousness in this relationship.

**Methods:**

The participants were 590 college students (272 males; mean age = 19.67) who completed a psychosocial battery, including the international physical activity questionnaire—short form (IPAQ-SF), the connor - davidson resilience scale (CD-RISC), the interaction anxiousness scale (IAS), and the mobile phone addiction index (MPAI). Correlations of variables were computed using Pearson’s test. Mediation models were tested using SPSSS PROCESS macro with the regression bootstrapping method.

**Results:**

PA were negatively associated with MPA behavior (r=-.21, p < 0.01). Resilience and interaction anxiousness moderated the relationship between PA and MPA. More importantly, PA could also influence MPA through the chain-mediating effects of resilience and interaction anxiousness.

**Conclusion:**

It is essential to improve resilience and reduce interaction anxiousness to reduce MPA problems through regular engagement in PA among college students.

## 1 Introduction

Mobile phone addiction, also known as MPA, is a type of addictive behavior that is linked to excessive usage of mobile phones (1, 2). It is characterized by individuals having a strong desire for and dependency on their mobile devices, causing them to partake in activities that involve the overuse of these devices (3, 4). Current research report that adolescents and young adults are an important group of mobile phone users and have a high prevalence of MPA (5, 6). The harmful effects of MPA are particularly prominent among college students, who may experience a variety of symptoms such as headaches, dizziness, body aches, numbness in the extremities, dry eyes, blurred vision, and mental health disorders like depression, social anxiety, stress, and insomnia (7–11). Experts specializing in mental health believe that MPA will become one of the most prevalent forms of technology addiction in the 21st century (12). Therefore, it is crucial to identify the risk and protective factors that contribute to MPA in order to prevent and manage this issue effectively.

Research has indicated that the development of MPA behavior is associated with various adverse consequences, including detrimental mental and physical well-being, academic underachievement, and interpersonal difficulties (13, 14). Specifically, individuals at the college level often encounter significant challenges in their daily lives, such as interpersonal and academic hurdles. Due to their limited self-control capabilities, they experience negative emotions when confronted with these challenges (15–17). In the presence of negative moods, college students tend to resort to engaging in MPA behavior as a means of regulating their emotions and obtaining immediate rewards. The use of mobile phones, being one of the most convenient and accessible electronic devices, provides an instant solution to fulfill multiple needs and serves as an easily accessible method for stress relief, thereby contributing to the emergence of MPA behavior among college students (18, 19).

The recommended amount of physical activity (PA) by the World Health Organization is at least 150 minutes per week for moderate-to-intense PA. Alternatively, engaging in vigorous to intense PA for more than 75 minutes each week is also advised. This is to prevent chronic diseases, as regular PA offers significant benefits for both physical and mental health (20). Several studies have found negative correlations between PA and MPA (21, 22). Recent research has indicated that PA can effectively predict MPA in a negative manner. Additionally, the potential mediating role of self-control between PA and MPA has been taken into consideration (23). These findings underscore the potential advantages of PA, which could serve as an important protective mechanism against MPA.

Resilience, defined by the American Psychological Association (2014), refers to “the ability to adapt well in the face of hardship, trauma, adversity, significant sources of stress, or even tragedy” (24). In simpler terms, resilience allows individuals to return to a previous state of normalcy or health following a trauma, accident, tragedy, or illness, which is vital for both mental and physical well-being (25). It is worth mentioning that engaging in regular physical activity (PA) has been scientifically proven to effectively enhance resilience among college students. This improvement in resilience subsequently boosts their problem-solving skills, self-confidence, and ability to regulate emotions (26, 27). According to the ego-depletion model of self-regulation, individuals who struggle to successfully adapt to social issues may experience reduced self-control when it comes to using their phones (28). Consequently, it can be inferred that college students with low levels of resilience may excessively rely on the internet as a coping mechanism when faced with challenges that they are unable to resolve, particularly social problems (29). Building upon this existing evidence, resilience can be considered an internal mechanism that mediates the relationship between PA and MPA.

Interaction anxiousness, also known as social anxiety, is a psychological condition characterized by an individual’s persistent fear and emotional discomfort in social or performance situations. Individuals experiencing interaction anxiousness often seek to escape or avoid association with other people (30). Embodied cognition theories suggest a close connection between our mental states and the physical situations we find ourselves in (31). Research has consistently shown that regular physical activity (PA) can lead to higher emotional stability and reduced anxiety (32–35). Additionally, the compensatory Internet use theory proposes that individuals who face psychological challenges in the real world may turn to the Internet or smartphones as a means of escape and pain reduction (36). Notably, individuals with higher levels of interaction anxiety tend to seek support from the Internet, leading to excessive use of mobile phones (37). Taking into account this scientific evidence, it is plausible to consider interaction anxiousness as a significant mediating factor in the relationship between PA and mobile phone addiction (MPA) among college students.

According to the dynamic model of psychological resilience, resilience can be considered a protective factor against negative emotions (e.g., interaction anxiousness) (38), which can be improved by regular physical exercise (39, 40). In other words, individuals with a higher level of resilience could more easily restore their mental balance while experiencing psychological distress, accompanied by a reduction in anxiety levels. Although we hypothesized that resilience and interaction anxiousness may both mediate the effect of PA on MPA, sequential mediation models may be based on the relationship between resilience and interaction anxiousness.

Based on the theories and literature, it appears to be a negative association between PA and MPA among college students in daily life. In sporadic research that has explored the underlying mechanisms between PA and MPA, it is suggested that resilience and interaction anxiousness play a mediating role during the process. The present study was guided by the following hypotheses:

(1) Resilience and interaction anxiousness mediate the relationship between PA and MPA in college students (H1→H5; H4→H3).
(2) Resilience and interaction anxiousness play a chain-mediating role in the relationship between PA and MPA among college students (H1→H2→H3).

**Figure 1:**
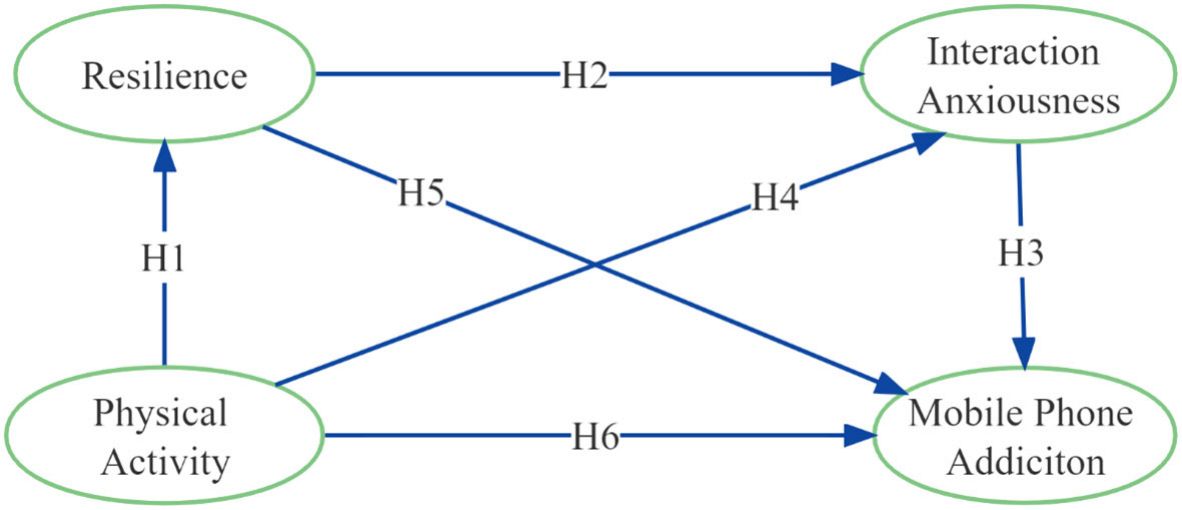
Research model.

This study followed these assumptions to investigate the relationships between the four key variables using the research hypothesis model shown in **Figure 1**.

## 2 Method

### 2.1 Participants

A convenience sampling approach was used to recruit targeted college students aged between 18 and 25 years in three university in Guangdong province, southern China. Eligible participants were described in the study and were asked whether they were willing to participate. After providing written informed consent, the participants were asked to complete a comprehensive questionnaire to collect demographic information and other subsequent procedures. Questionnaires were collected from March 2022 to May 2022, and the initial sample comprised 647 college students. After the questionnaires were collected, we conducted repeated inspections and screenings to eliminate questionnaires that took less than 2 min to complete, had irregular answers, and were logically contradictory. The final sample of valid responses was obtained from 590 participants (272 males and 318 females; Mage = 19.67, SD = 1.48). The recruitment and data collection procedures were approved by the Ethics Committee of Shenzhen University.

### 2.2 Measure

#### 2.2.1 Physical activity

The levels of PA among college students were evaluated through the utilization of the validated Chinese version (41) of the international questionnaire on physical activity—abbreviated form (IPAQ-SF) (42). IPAQ-SF determines the PA levels in the past 7 days by gathering and dividing the time spent on vigorous activity, moderate activity, walking, and sitting. The total PA level (represented by metabolic equivalent [MET]) was calculated based on the quantities of vigorous, moderate, and walking activities. In this study, Cronbach’s alpha for the IPAQ-SF was determined to be 0.87.

#### 2.2.2 Resilience

To measure resilience over the last month, we utilized the Chinese edition of the Connor-Davidson resilience scale (CD-RISC) (35). This scale, consisting of 25 items, incorporates three main aspects: tenacity, strength, and optimism. Participants rated each item on a 5-point Likert scale, ranging from 1 (hardly ever) to 5 (almost always). A greater score indicates a higher degree of resilience. Our study observed a high level of internal consistency (Cronbach’s α = 0.93).

#### 2.2.3 Interaction anxiousness

In order to evaluate the levels of interaction anxiousness, the Chinese adaptation of the interaction anxiousness scale (IAS) was employed (43). Consisting of 15 items, this version requires participants to rate their responses on a 5-point Likert scale. Respondents rate the items on a scale ranging from 1 (never) to 5 (always). Enhanced scores signify elevated levels of interaction anxiety. The reliability of the IAS, as measured by Cronbach’s alpha, was found to be 0.89 in the current study.

#### 2.2.4 Mobile phone addiction

To evaluate mobile phone addiction, researchers utilized the mobile phone addiction index (MPAI) (44). This index comprises 17 items that gauge four different aspects of smartphone addiction: lack of control over cravings, anxiety levels, disorientation and withdrawal, and diminished productivity. Survey respondents provided answers to these items using a 5-point scale, ranging from 1 (never) to 5 (always). Prior investigations have attested to the MPAI’s reliability and validity among Chinese adolescents and young adults (45). In our current study, the measure displayed strong internal consistency, as indicated by Cronbach’s α coefficient of 0.88.

### 2.3 Data analysis

Data were analyzed using SPSS 21.0 software and the PROCESS macro (44). Specifically, the total score of each scale was first calculated according to the corresponding formula and rules. Second, Harman’s single-factor test was used to assess common method bias, which indicated a concern if one factor explained more than 50% of the total variance (46, 47). Third, gender differences in all tested variables were tested by independent sample t-test. Pearson’s correlations between each of the two dependent variables (PA, resilience, interaction anxiousness, and MPA) were tested to support the research hypotheses. Finally, according to our hypotheses, we constructed three models to test for validity using SPSS 21.0 (Model 6, the PROCESS macro). The bias-corrected percentile bootstrap method was used to test the mediation models (mediation model of resilience; mediation model of interaction anxiousness) and the chain mediation model of resilience and interaction anxiousness. The mediation model was implemented with 5,000 bootstrap samples and 95% corrected confidence intervals (CIs) (48, 49).

## 3 Results

### 3.1 Common method bias

Common method bias was tested using Harman’s single-factor method. We first conducted exploratory factor analyses for all items and each scale. The results indicated that 11 factors had eigenvalues higher than 1.0; the first factor explained only 20.74% of the total variance, which is lower than 50%. Thus, the results indicated that the common method bias was not large enough to distort the results.

### 3.2 Descriptive statistics

Descriptive statistics showed that the scores of self-reported levels of PA, resilience, interaction anxiousness, and MPA were moderate (**Table 1**). Moreover, resilience and interaction anxiousness showed significant gender differences; specifically, male students demonstrated higher resilience scores than female students, and female students had higher interaction anxiousness than male students. Pearson’s correlation analysis found that PA had a significantly positive correlation with resilience, and it was negatively correlated with interaction anxiety and MPA. Resilience was positively associated with interaction anxiousness and MPA, and interaction anxiousness had a significantly positive association with MPA. Correlation coefficients are displayed in **Table 2**.

**Table 1:** Gender difference in all tested variables.

Variables	Total (n = 590)	Male (n = 272)	Female (n = 318)	T	P
	M ± SD	M ± SD	M ± SD		
Age	19.67 ± 1.48	19.95 ± 1.70	19.44 ± 1.22	4.16**	0.00
Physical Activity (MET)	3696.46 ± 2295.19	3683.254 ± 2281.37	3707.76 ± 2310.48	-0.13	0.90
Resilience	77.25 ± 20.35	79.23 ± 19.823	75.55 ± 20.67	2.197*	0.03
Interaction Anxiousness	42.47 ± 10.21	42.56 ± 10.26	45.1 ± 10.04	-3.039**	0.00
Mobile Phone Addiction	42.47 ± 10.56	42.19 ± 9.75	42.71 ± 11.22	-0.59	0.55

*: P < 0.05, **: P < 0.01

**Table 2:** Correlations of all tested variables.

-	Physical Activity (MET)	Resilience	Interaction Anxiousness	Mobile Phone Addiction
Physical Activity (MET)	–	–	–	–
Resilience	-.30**	–	–	–
Interaction Anxiousness	.23**	-.31**	–	–
Mobile Phone Addiction	-.21**	.31**	.22**	–

*: P < 0.05, **: P < 0.01

### 3.3 Chained mediating analyses

**Figure 2:**
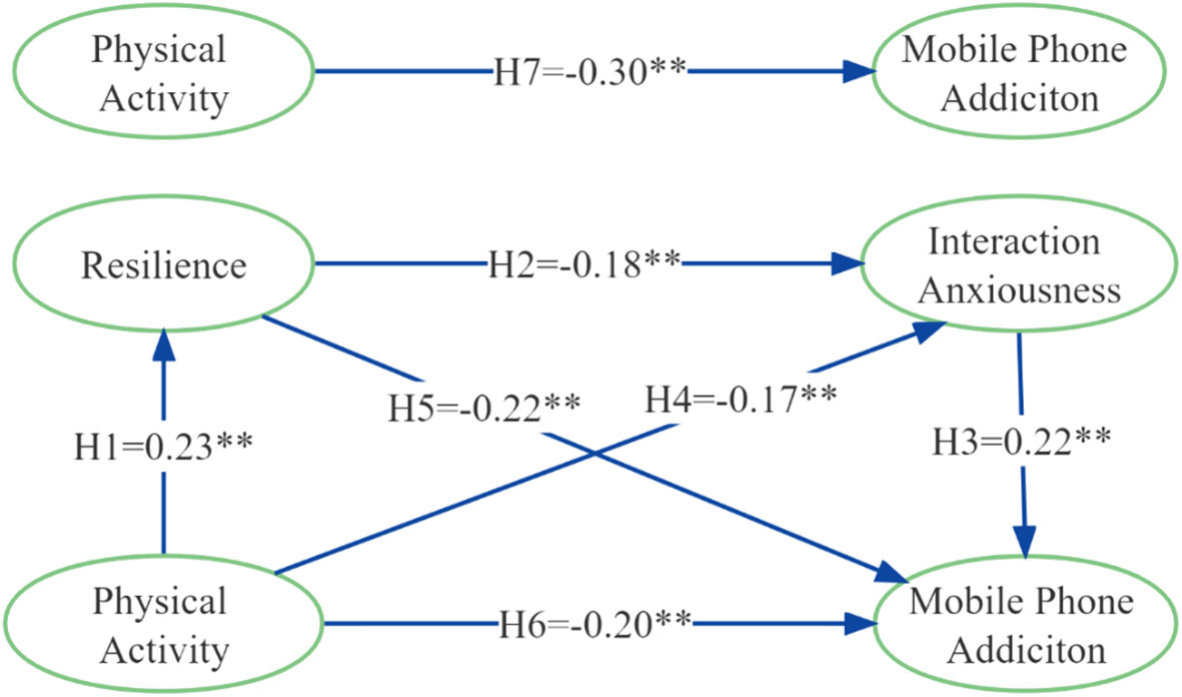
Regression analysis of the chain mediating model. *: *P* < 0.05, **: *P* < 0.01.

According to the model 6 in the Process program, a chain mediation model was established with PA as an independent variable, resilience and interaction anxiousness as mediating variables, and MPA as a dependent variable. As shown in **Figure 2**, PA significantly and positively predicted resilience (H1 = 0.23, *P* < 0.01), resilience significantly negatively predicted interaction anxiousness (H2 = -0.18, *P* < 0.01), and interaction anxiousness significantly and positively predicted MPA (H3 = 0.22 P *<*0.01).

**Table 3** showed the overall path coefficients of the mediation analysis. Specifically, in the total effect model, the direct predictive effect of PA on MPA in this study revealed that PA was able to predict MPA significantly and negatively (H7 = -0.30, 95%CIs [-0.002, -0.001]). When resilience and interaction anxiousness as mediating variables were added to construct a chain mediation model, PA still played a significant role in predicting MPA (H6 = -0.20, 95%Cls [-0.002, -0.001]). The indirect effect of perceived social support through resilience and interaction anxiousness was also significant (H1→H2→H3 = -0.01, 95% CIs [-0.016, -0.003]), which confirmed the research hypotheses. Each mediation path had a significant effect on MPA (H1→H5, H4→H3= -0.05, -0.04, 95% CIs [-0.075, -0.029], [-0.063, -0.018], respectively), which also confirmed the research hypotheses.

**Table 3:** Chain mediation analysis results.

		Standardized Effect	Standard Error	95% Confidence Interval	
				Lower	Upper
H7	Total effect	-0.30	0.000	-0.002	-0.001
H6	Direct effect	-0.20	0.000	-0.002	-0.001
H1→H5	Path 1	-0.05	0.012	-0.075	-0.029
H4→H3	Path 2	-0.04	0.012	-0.063	-0.018
H1→H2→H3	Path 3	-0.01	0.003	-0.016	-0.004

## 4 Discussion

The purpose of this study was to analyze the effect of PA on MPA among college students and the mediating role of resilience and interaction anxiousness in this relationship, which further extends previous studies on the mechanism of PA on MPA and helps to gain a better understanding of the potential benefit of PA concerning MPA (47). Three mediating paths were found in the mechanism underlying the relationship between PA and MPA among Chinese college students. Specifically, resilience and interaction anxiousness moderated the relationship between PA and MPA. More importantly, PA could also influence MPA through the chain-mediating effects of resilience and interaction anxiousness. Physical exercise is a positive and effective means of health promotion, and an increase in exercise can improve the brain’s emotional processing ability to relieve anxiety and reduce phone addiction. Thus, based on the above mentioned mechanisms, physical exercise could be an intervention to treat college students with MPA.

First, resilience appears to be an important mediator in the effects of PA on MPA among college students. College students with higher levels of PA had higher levels of resilience, which significantly and positively predicted MPA among them. Our study found that PA has a significant effect on resilience, similar to the findings of previous studies (50, 51), because it can enhance emotion regulation by protecting neurons in regions of the brain, such as the striatum and hippocampus (27). Besides, according to the dynamic model of psychological resilience, when college students face adverse events such as bad interpersonal relationships and academic pressure, individuals with high resilience levels regulate their negative emotions and avoid being frustrated by real problems and indulging in the online world (51). The university stage is a transition period into social life, where many stressors and challenges are experienced in daily life and studies. Participation in PA may be an appropriate way to regulate emotions by enhancing psychological resilience, resulting in reduced MPA.

Second, it appears that interaction anxiousness mediated the association between PA and MPA among college students. Due to the mature entertainment and social functions of cell phones, as well as their universality and accessibility, their use has become the main way for college students to satisfy the need for interaction while reducing the anxiety of realistic interactions. The final result is that college students indulge more in the virtual environment built by cell phones, thus increasing their MPA level (52). The interaction of the person-affect-cognition-execution (I-PACE) model suggests that individuals’ characteristics (e.g., personality, spirit) may influence their cognitive function and behaviors; thus, reducing negative emotions through regular PA is a potential mechanism for reducing MPA (53). As known, exercise stimulates the release of endorphins, a kind of polypeptide beneficial for reducing negative emotions (47). From a physiological point of view, increased exercise can improve the occurrence of anxiety, which reduces the emotional relief of mobile phone use. Furthermore, it is important to acknowledge that consistent engagement in physical activity can facilitate the establishment of new social connections. These newfound relationships may continue to be significant post-exercise, providing emotional support and aids in reducing anxiety (54). Additionally, these social interactions may lead to decreased reliance on mobile devices by participating in other activities (e.g., Watch a movie, reading) (55), ultimately lowering levels of problematic mobile phone usage.

Third, this study found that resilience and interaction anxiousness played a chain-mediating role in the association between PA and MPA among college students, revealing the mechanism by which PA affects MPA through the combined effect of resilience and interaction anxiousness. Our findings suggest close connections between resilience and interaction anxiousness, which echoes a previous study of negative emotions (35). Based on our findings, we speculate that the higher the PA level of college students, the higher the level of resilience, and the more the benefits of flexibly dealing with the negative effects of changes in the external environment, such as reducing social avoidance and distress in social situations, which can reduce the tendency toward cell phone addiction (56).

## 5 Practical implications and limitations

In summary, the results support the hypotheses of our study, and this study introduced two variables – resilience and interaction anxiousness – which expands on the existing research into the impact of PA on MPA. Following the PA Guidelines for World Health Organization explanation, we suggest the actual amount of PA can higher than the PA Guidelines for World Health Organization recommend because it can bring more mental health benefits. In particular, during the COVID-19 epidemic, periods of confinement can lead to physical dysfunction and mental distress (e.g., MPA), partially attributed to reductions in habitual physical activity. Our findings indicate that college students should actively perform PA to improve resilience and reduce interaction anxiousness, which is conducive to reducing the negative consequences of MPA. Our finding have certain guiding significance for the prevention of MPA behavior among college students. Therefore, college students need to closely monitor their own the level of PA during the COVID-19 epidemic, especially for those who want to regulate their own mobile phone use behaviors.

However, some limitations of this study should be mentioned when interpreting its results. First, the data collected were cross-sectional and could only reveal a correlation—and not a causal relationship—between PA and MPA among college students. Further research and experiments are needed to verify this causality. Second, all subjects in this study were from one district of Guangdong province, China, which limited the representativeness of the sample. Extending the survey to a national sample is necessary for future studies. Third, all data in this study were collected through self-reported scales, and bias may exist because of the social desirability effect and/or memory errors. Finally, from the perspective of data analysis, gender differences were observed in resilience and interaction anxiety. More research is thus needed to uncover the mechanisms underlying the association between PA and MPA.

## 6 Conclusion

In this study, we found that PA was significantly related to MPA among college students and that this relationship was mediated by a series of associations between resilience and interaction anxiety. It is thus recommended to improve resilience and reduce interaction anxiousness to curb MPA problems through regular engagement in PA among college students.

## Data availability statement

The raw data supporting the conclusions of this article will be made available by the authors, without undue reservation.

## Ethics statement

Ethical approval for the study was obtained from the Ethics Committee of Shenzhen University. Informed consent to participate was obtained for all participants before survey initiation. All methods were per−formed in accordance with the relevant guidelines and regulations.

## Author contributions

JW: Conceptualization, Data curation, Methodology, Project administration, Writing – original draft. WX: Conceptualization, Data curation, Writing – original draft. BL: Data curation, Writing – review & editing. JY: Writing – review & editing. KZ: Writing – review & editing. QS: Writing – review & editing. ZR: Writing – original draft, Writing – review & editing.

## References

[1] Yang XJ Liu QQ Lian SL Zhou ZK. Are bored minds more likely to be addicted? The relationship between boredom proneness and problematic mobile phone use. Addictive Behav. (2020) 108:106426. doi: 10.1016/j.addbeh.2020.106426 32446143

[2] Zhang G Yang X Tu X Ding N Lau JTF. Prospective relationships between mobile phone dependence and mental health status among Chinese undergraduate students with college adjustment as a mediator. J Affect Disord. (2020) 260:498–505. doi: 10.1016/j.jad.2019.09.047 31539686

[3] Zou Z Wang H d’Oleire Uquillas F Wang X Ding J Chen H. Definition of substance and non-substance addiction. Subst Non-substance Addict. (2017), 21–41. doi: 10.1007/978-981-10-5562-1_2 29098666

[4] Billieux J Maurage P Lopez-Fernandez O Kuss DJ Griffiths MD. Can disordered mobile phone use be considered a behavioral addiction? An update on current evidence and a comprehensive model for future research. Curr Addict Rep. (2015) 2:156–62. doi: 10.1007/s40429-015-0054-y

[5] Hong FY Chiu SI Huang D-H. A model of the relationship between psychological characteristics, mobile phone addiction and use of mobile phones by Taiwanese university female students. Comput Hum Behav. (2012) 28:2152–9. doi: 10.1016/j.chb.2012.06.020

[6] Lopez-Fernandez O Kuss DJ Romo L Morvan Y Kern L Graziani P. Self-reported dependence on mobile phones in young adults: A European cross-cultural empirical survey. J Behav Addict. (2017) 6:168–77. doi: 10.1556/2006.6.2017.020 PMC5520117 28425777

[7] Sahu M Gandhi S Sharma MK. Mobile phone addiction among children and adolescents: A systematic review. J Addict Nurs. (2019) 30:261–8. doi: 10.1097/JAN.0000000000000309 31800517

[8] Višnjić A Veličković V Sokolović D Stanković M Mijatović K Stojanović M. Relationship between the manner of mobile phone use and depression, anxiety, and stress in university students. Int J Environ Res Public Health. (2018) 15:697. doi: 10.3390/ijerph15040697 29642471 PMC5923739

[9] Ivanova A Gorbaniuk O Błachnio A Przepiórka A Mraka N Polishchuk V. Mobile phone addiction, phubbing, and depression among men and women: a moderated mediation analysis. Psychiatr Q. (2020) 91:655–68. doi: 10.1007/s11126-020-09723-8 PMC7395043 32146681

[10] Zhang T Gong N Jia R Li H Ni X. Stroop effect in smartphone addiction among college students. Medicine. (2021) 100(30):e26741. doi: 10.1097/MD.0000000000026741 34397714 PMC8322516

[11] Kaya F Bostanci Daştan N Durar E. Smart phone usage, sleep quality and depression in university students. Int J Soc Psychiatry. (2021) 67:407–14. doi: 10.1177/0020764020960207 32969293

[12] Lin YH Lin SH Yang CCH Kuo TBJ. Psychopathology of everyday life in the 21st century: smartphone addiction. In: Internet Addiction. Springer (2017). p. 339–58.

[13] Li L Xu DD Chai JX Wang D Li L Zhang L. Prevalence of Internet addiction disorder in Chinese university students: A comprehensive meta-analysis of observational studies. J Behav Addict. (2018) 7:610–23. doi: 10.1556/2006.7.2018.53 PMC6426360 30010411

[14] Deci EL Ryan RM. The” what” and” why” of goal pursuits: Human needs and the self-determination of behavior. psychol Inq. (2000) 11:227–68. doi: 10.1207/S15327965PLI1104_01

[15] Augner C Hacker GW. Associations between problematic mobile phone use and psychological parameters in young adults. Int J Public Health. (2012) 57:437–41. doi: 10.1007/s00038-011-0234-z 21290162

[16] Kuang-Tsan C Fu-Yuan H. Study on relationship among university students’ life stress, smart mobile phone addiction, and life satisfaction. J Adult Dev. (2017) 24:109–18. doi: 10.1007/s10804-016-9250-9

[17] Lepp A Barkley JE Karpinski AC. The relationship between cell phone use, academic performance, anxiety, and satisfaction with life in college students. Comput Hum Behav. (2014) 31:343–50. doi: 10.1016/j.chb.2013.10.049

[18] Fu L Wang P Zhao M Xie X Chen Y Nie J. Can emotion regulation difficulty lead to adolescent problematic smartphone use? A moderated mediation model of depression and perceived social support - ScienceDirect. Child Youth Serv Rev (2020) 108:104660. doi: 10.1016/j.childyouth.2019.104660

[19] Southwick SM Ozbay F Mayes LC. Psychological and biological factors associated with resilience to stress and trauma. Lanham, MD, US: Jason Aronson (2008).

[20] World Health, O. Public health implications of excessive use of the internet, computers, smartphones and similar electronic devices: Meeting report, Main Meeting Hall, Foundation for Promotion of Cancer Research, National Cancer Research Centre, Tokyo, Japan, 27-29 August 2014. World Health Organization (2015).

[21] Kim S-E Kim J-W Jee Y-S. Relationship between smartphone addiction and physical activity in Chinese international students in Korea. J Behav Addict. (2015) 4:200–5. doi: 10.1556/2006.4.2015.028 PMC4627682 26551911

[22] Zagalaz-Sánchez ML Cachón-Zagalaz J Sánchez-Zafra M Lara-Sánchez A. Mini review of the use of the mobile phone and its repercussion in the deficit of physical activity. Front Psychol. (2019) 10:1307. doi: 10.3389/fpsyg.2019.01307 31244720 PMC6563677

[23] Guo Kl Ma Qs Yao Sj Liu C Hui Z Jiang J. The relationship between physical exercise and mobile phone addiction tendency of university students in China: A moderated mediation model. Front Psychol. (2022) 13. doi: 10.3389/fpsyg.2022.730886 PMC8884265 35237204

[24] Cousins JN Fernández G. The impact of sleep deprivation on declarative memory. Prog Brain Res. (2019) 246:27–53. doi: 10.1016/bs.pbr.2019.01.007 31072562

[25] Babić R Babić M Rastović P Ćurlin M Šimić J Mandić K. Resilience in health and illness. Psychiatria Danubina. (2020) 32:226–32. 32970640

[26] Xia B Ma Z Hu Y. Research on the relationship between physical exercise, psychological flexibility and positive emotion of college students based on computer mathematical model. J Phys: Conf Ser. (2020) 1578(1):012009. doi: 10.1088/1742-6596/1578/1/012009

[27] Xu S Liu Z Tian S Ma Z Jia C Sun G. Physical activity and resilience among college students: The mediating effects of basic psychological needs. Int J Environ Res Public Health. (2021) 18:3722. doi: 10.3390/ijerph18073722 33918303 PMC8038173

[28] Tokunaga RS Rains SA. An evaluation of two characterizations of the relationships between problematic Internet use, time spent using the Internet, and psychosocial problems. Hum Communication Res. (2010) 36:512–45. doi: 10.1111/j.1468-2958.2010.01386.x

[29] Peng W Liu M. Online gaming dependency: a preliminary study in China. Cyberpsychol Behavior Soc Networking. (2010) 13:329–33. doi: 10.1089/cyber.2009.0082 20557254

[30] Buyukbayraktar CG. Predictive relationships among smartphone addiction, fear of missing out and interaction anxiousness. Eur J Educ Sci. (2020) 7:1–16. doi: 10.19044/ejes.v7no2a1

[31] Egger SW Remington ED Chang CJ Jazayeri M. Internal models of sensorimotor integration regulate cortical dynamics. Nat Neurosci. (2019) 22:1871–82. doi: 10.1038/s41593-019-0500-6 PMC6903408 31591558

[32] Qian HB Wang ZY. Literature Review of Physical Exercise and Mental Health Study. Nanjing, China: China School Physical Education (Higher Education (2017).

[33] Di Benedetto M Len Burns G Lindner H Kent S. A biopsychosocial model for depressive symptoms following acute coronary syndromes. Psychol Health. (2010) 25:1061–75. doi: 10.1080/08870440903019535 20204970

[34] Vasey MW Bosmans G Ollendick TH. The developmental psychopathology of anxiety. In: Handbook of Developmental Psychopathology. US: Springer (2014). p. 543–60.

[35] Schuch FB Bulzing RA Meyer J Vancampfort D Firth J Stubbs B. Associations of moderate to vigorous physical activity and sedentary behavior with depressive and anxiety symptoms in self-isolating people during the COVID-19 pandemic: A cross-sectional survey in Brazil. Psychiatry Res. (2020) 292:113339. doi: 10.1016/j.psychres.2020.113339 32745795 PMC7384423

[36] Kardefelt-Winther D. A conceptual and methodological critique of internet addiction research: Towards a model of compensatory internet use. Comput Hum Behav. (2014) 31:351–4. doi: 10.1016/j.chb.2013.10.059

[37] Bian M Leung L. Linking loneliness, shyness, smartphone addiction symptoms, and patterns of smartphone use to social capital. Soc Sci Comput Rev. (2015) 33:61–79. doi: 10.1177/0894439314528779

[38] Li X Yu H Yang N. The mediating role of resilience in the effects of physical exercise on college students’ negative emotions during the COVID-19 epidemic. Sci Rep. (2021) 11:1–8. doi: 10.1038/s41598-021-04336-y 34972833 PMC8720086

[39] Spies G Seedat S. Depression and resilience in women with HIV and early life stress: does trauma play a mediating role? A cross-sectional study. BMJ Open. (2014) 4:e004200. doi: 10.1136/bmjopen-2013-004200 PMC3939658 24566532

[40] Richardson GE. The metatheory of resilience and resiliency. J Clin Psychol. (2002) 58:307–21. doi: 10.1002/jclp.10020 11836712

[41] Wang C Chen P Zhuang J. Validity and reliability of international physical activity questionnaire–short form in Chinese youth. Res Q Exercise Sport. (2013) 84:S80–6. doi: 10.1080/02701367.2013.850991 24527570

[42] Meeus M Van Eupen I Willems J Kos D Nijs JO. Is the International Physical Activity Questionnaire-short form (IPAQ-SF) valid for assessing physical activity in Chronic Fatigue Syndrome? Disability Rehabil. (2011) 33:9–16. doi: 10.3109/09638288.2010.483307 20446802

[43] Leary MR. Social anxiousness: The construct and its measurement. J Pers Assess. (1983) 47:66–75. doi: 10.1207/s15327752jpa4701_8 6834234

[44] Leung L. Leisure boredom, sensation seeking, self-esteem, and addiction: Symptoms and patterns of cell phone use. In: Mediated interpersonal communication. Routledge (2008). p. 373–96.

[45] Lian L You X Huang J Yang R. Who overuses smartphones? Roles of virtues and parenting style in smartphone addiction among Chinese college students. Comput Hum Behav. (2016) 65:92–9. doi: 10.1016/j.chb.2016.08.027

[46] Podsakoff PM MacKenzie SB Lee J-Y Podsakoff NP. Common method biases in behavioral research: a critical review of the literature and recommended remedies. J Appl Psychol. (2003) 88:879. doi: 10.1037/0021-9010.88.5.879 14516251

[47] Akdoğan R Çimşir E. Linking inferiority feelings to subjective happiness: Self-concealment and loneliness as serial mediators. Pers Individ Dif. (2019) 149:14–20. doi: 10.1016/j.paid.2019.05.028

[48] Hayes AF. PROCESS: A versatile computational tool for observed variable mediation, moderation, and conditional process modeling. (2012). Retrieved from http://www.afhayes.com/public/process2012.pdf.

[49] Bolin JH. Introduction to mediation, moderation, and conditional process analysis: a regression-based approach. (2014) p. 335–337.

[50] Zschucke E Renneberg B Dimeo F Wüstenberg T Ströhle A. The stress-buffering effect of acute exercise: Evidence for HPA axis negative feedback. Psychoneuroendocrinology. (2015) 51:414–25. doi: 10.1016/j.psyneuen.2014.10.019 25462913

[51] Furlong MJ Ritchey KM O’Brennan LM. Developing norms for the California Resilience Youth Development Module: Internal assets and school resources subscales. California School Psychol. (2009) 14:35–46. doi: 10.1007/BF03340949

[52] Mimi MY Tang SK Wan VTC Vong SKS. The effectiveness of physical exercise training in pain, mobility, and psychological well-being of older persons living in nursing homes. Pain Manage Nurs. (2014) 15:778–88. doi: 10.1016/j.pmn.2013.08.003 24361207

[53] Brand M Young KS Laier C Wölfling K Potenza MN. Integrating psychological and neurobiological considerations regarding the development and maintenance of specific Internet-use disorders: An Interaction of Person-Affect-Cognition-Execution (I-PACE) model. Neurosci Biobehav Rev. (2016) 71:252–66. doi: 10.1016/j.neubiorev.2016.08.033 27590829

[54] Sawka KJ McCormack GR Nettel-Aguirre A Hawe P Doyle-Baker PK. Friendship networks and physical activity and sedentary behavior among youth: a systematized review. Int J Behav Nutr Phys Activity. (2013) 10:130. doi: 10.1186/1479-5868-10-130 PMC4220781 24289113

[55] Monteiro D Rodrigues F Lopes VP. Social support provided by the best friend and vigorous-intensity physical activity in the relationship between perceived benefits and global self-worth of adolescents. Rev Psicodidáctica (English ed.). (2021) 26:70–7. doi: 10.1016/j.psicod.2020.11.004

[56] Ma X Wang Y Hu H Tao XG Zhang Y Shi H. The impact of resilience on prenatal anxiety and depression among pregnant women in Shanghai. J Affect Disord. (2019) 250:57–64. doi: 10.1016/j.jad.2019.02.058 30831542

